# Catheter Ablation of Idiopathic Premature Ventricular Contractions and Ventricular Tachycardias Originating from Right Ventricular Septum

**DOI:** 10.1371/journal.pone.0067038

**Published:** 2013-06-25

**Authors:** Wu Lian-Pin, Li Yue-Chun, Zhao Jing-Lin, Zheng Cheng, Chen Jun-Hua, Hong Jun, Lin Jia-Xuan, Li Jin, Li Jia, Ji Kang-Ting, Lin Jia-Feng

**Affiliations:** Department of Cardiology, Second Affiliated Hospital of Wenzhou Medical College, Wenzhou, China; Medical University Innsbruck, Austria

## Abstract

**Background:**

Idiopathic premature ventricular contractions (PVCs) and ventricular tachycardias (IVTs) originating from the subtricuspid septum and near the His bundle have been reported. However, little is known about the prevalence, distribution, electrocardiographic characteristics and the efficacy of radiofrequency catheter ablation (RFCA) for the ventricular arrhythmias arising from the right ventricular (RV) septum. This study aimed to investigate electrocardiographic characteristics and effects of RFCA for patients with symptomatic PVCs/IVTs, originating from the different portions of the RV septum.

**Methodology/Principal Findings:**

Characteristics of body surface electrocardiogram and electrophysiologic recordings were analyzed in 29 patients with symptomatic PVCs/IVTs originating from the RV septum. Among 581 patients with PVCs/IVTs, the incidence of ventricular arrhythmias originating from the RV septum was 5%. Twenty (69%) had PVCs/IVTs from the septal portion of the tricuspid valvular RV region (3 from superoseptum, 15 from midseptum, 2 from inferoseptum), and 9 (31%) from the septal portion of the basal RV (1 from superoseptum, 4 from midseptum, 4 from inferoseptum). There were different characteristics of ECG of PVCs/VT originating from the different portions of the RV septum. Twenty-seven of 29 patients with PVCs/IVTs arising from the RV septum were successfully ablated (93.1% acute success).

**Conclusions/Significance:**

ECG characteristics of PVCs/VTs originating from the different portions of the RV septum are different, and can help regionalize the origin of these arrhythmias. The septal portion of the tricuspid valvular RV region was the preferential site of origin. RFCA was effective and safe for the PVCs/IVTs arising from the RV septum.

## Introduction

The majority of idiopathic right ventricular arrhythmias (VAs), including idiopathic premature ventricular contractions (PVCs) and ventricular tachycardias (IVTs), originate from the right ventricular outflow tract (RVOT), with a small part of them originating from the inflow, free wall, or apex of the right ventricle [1]–[6]. Idiopathic PVCs and VTs originating from the right ventricular (RV) septum are rare. Only few cases of idiopathic VAs have been reported to originate from the subtricuspid septum and near the His bundle [7]–[10]. However, little is known about the prevalence, distribution, electrocardiographic (ECG) characteristics and the efficacy of radiofrequency catheter ablation (RFCA) for the VAs arising from the RV septum. The purpose of this study was to analyze the ECG characteristics and the outcome of catheter ablation for such PVCs/IVTs originating from the RV septum.

## Methods

### Study Population

From July 2006 to January 2012, a total of 581 patients (250 men and 331 women; age 46.9±17.2 years [mean±SD] ) without structural heart disease were presented for catheter ablation for PVCs/IVTs in our hospital. Twenty-nine of the 581 patients were found to have idiopathic PVCs/IVTs originating from the RV septum and are the focus of the present study. All patients were verified as having no structural heart disease, including coronary artery disease, valvular heart disease, congenital heart disease, left ventricle hypertrophy, and right ventricle abnormalities by routine biochemistry tests, X-ray, color echocardiography examination, exercise electrocardiogram testing, magnetic resonance imaging (MRI), and/or cardiac catheterization with coronary angiography or RV contrast angiography. Before RFCA, a 12-lead ECG was obtained, and 24 h of ambulatory ECG monitoring (Holter) was carried out at least once. The ECG was monitored for 24 h just before catheter ablation. The 29 patients with idiopathic PVCs/IVTs from the RV septum were compared with a randomly chosen series of 125 patients with idiopathic PVCs/IVTs in whom the site of origin was in the RVOT (n = 87, 56 women and 31 men; age 48.1±17.2 years) or the RV free wall (n = 38, 13 women and 25 men; age 36.2±17.7 years). A pace mapping study was also performed in 10 control subjects (6 women and 4 men; age 37.6±15.1 years) without structural heart disease after successful ablation of their original atrioventricular nodal reentrant tachycardia to determine the ECG characteristics of idiopathic PVCs/IVTs originating from the RV septum.

### Ethics Approval

Ethical approval was obtained from the Ethics Committee of the Second Affiliated Hospital of Wenzhou Medical College, and All participants consented to the experimental procedures. Written informed consent was obtained from each participant.

### Inclusion Criteria

The selection criteria of patients were the following reasons : (1) frequent or consecutive PVC occurrence, the average PVC count ≥10000/24 h; (2) inability of the patient to tolerate PVCs/IVTs or unsuccessful treatment with at least one antiarrhythmic drug; (3) no structural heart disease; and (4) consent for the catheter ablation procedure.

### Electrophysiologic Study and RFCA

Anti-arrhythmic drugs were withdrawn in all patients at least five half-lives before ablation, with the exception of amiodarone that was withdrawn eight weeks before intervention. Standard multielectrode catheters were inserted and positioned in the RV apex, RVOT, and His-bundle region through femoral veins under fluoroscopic guidance. A programmed electrical stimulation was performed from the RV apex and RVOT at basic drive cycle lengths of 600, 500, and 430 ms, delivering a maximum of three extrastimuli. If the clinical arrhythmia did not occur spontaneously and was not induced at baseline, intravenous isoproterenol (2–4 µg/min) was administered to induce arrhythmia. A 12-lead surface ECG was monitored and recorded on a multichannel oscilloscopic recorder**.** Detailed endocardial activation mapping and pace mapping were performed using an 8F quadripolar catheter with a deflectable tip and a 4-mm distal electrode. If the ventricular electrograms from the RV septum were the earliest of the entire RV endocardial mapping sites, we occasionally mapped the aortic valve cusps.

The target site for RFCA was determined by activation mapping (earliest local activation time preceding the earliest surface QRS by ≥20 msec) in patients with frequent PVCs/sustained IVT, and by pace mapping (≥11/12–lead concordance of major and minor deflections between the pace map and the clinical PVCs) in those with infrequent arrhythmia. After the target site was located, RFCA was applied in all patients by using irrigated-tip catheter (43°C, 30 W, 20 ml/min) with the three-dimensional mapping (Ensite NavX system or Carto XP or Carto 3 system) or a conventional catheter under temperature control (target temperature 55°C, and starting power of 20 W with gradual titration to a maximum power of 50 W). If the PVCs/IVTs terminated within 10 s of ablation, or if they increased in frequency within the initial 10 s of ablation, additional current was applied for another 60 to 180 s. If PVCs/IVTs did not terminate within 10 s, the radiofrequency energy application was terminated and another target site was sought. Acute procedural successful ablation was defined as complete elimination of spontaneous or inducible VAs. Programmed electrical stimulation was repeated at 30 min after the last application of radiofrequency energy to confirm the absence of inducible VAs before removing all catheters and sheaths. If the site demonstrating a perfect pace map was exactly where the largest His bundle potential was recorded, RF ablation could not be applied in order to avoid atrioventricular nodal block. If the best pace mapping site was close to the His bundle, the RF energy was cautiously delivered starting with a low power setting at the site that was at least 5 mm away from the recording site of the largest His bundle potential.

### Definition of PVCs/IVTs Originating from the RV Septum

**Figure 1:**
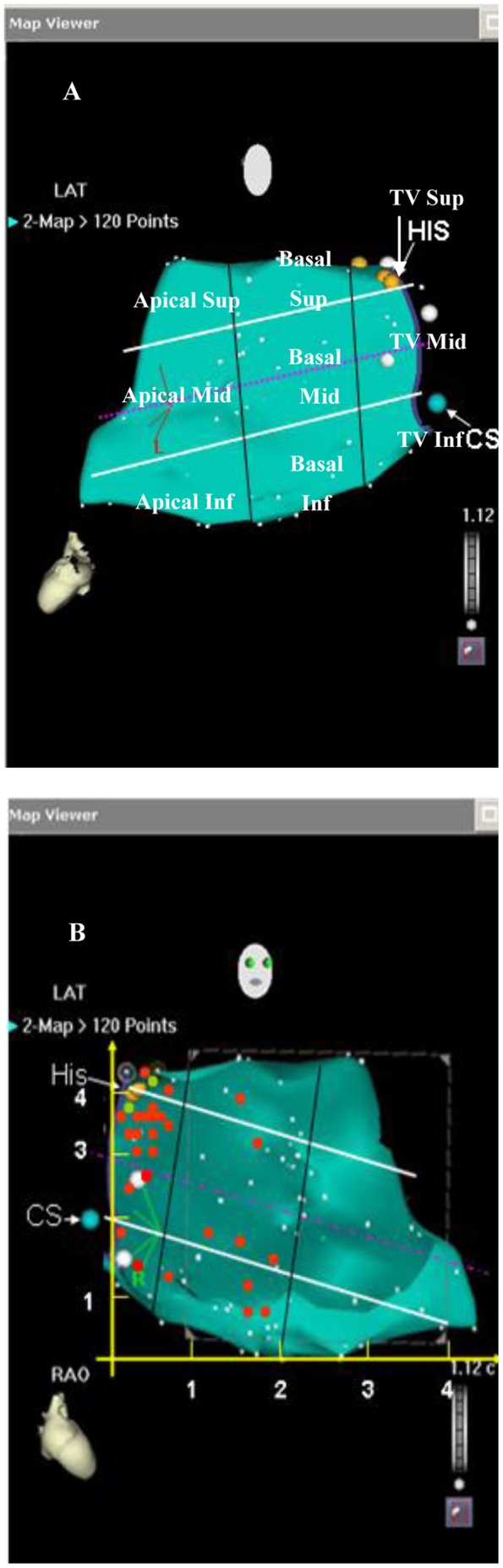
Schematic left lateral (A) and right anterior oblique (B) view of the right ventricular septum divided into nine regions and indicating the distribution of the origin of idiopathic ventricular arrhythmias, represented by red dots (successful ablation sites) and green dots (unsuccessful ablation sites). TV Sup: the superoseptal portion of the tricuspid valvular right ventricular region; TV Mid: the midseptal portion of the tricuspid valvular right ventricular region; TV Inf: the inferoseptal portion of the tricuspid valvular right ventricular region; Basal Sup: the superoseptal portion of the basal right ventricular region; Basal Mid: the midseptal portion of the basal right ventricular region; Basal Inf: the inferoseptal portion of the basal right ventricular region; Apical Sup: the superoseptal portion of the apical right ventricular region; Apical Mid: the midseptal portion of the apical right ventricular region; Apical Inf: the inferoseptal portion of the apical right ventricular region; His: largest His-bundle potential (yellow dot); CS: coronary sinus (blue dot).

To facilitate the identification of region-specific ECG features that might suggest an RV septum origin, sites of origin of PVCs/IVTs in the RV septum from top to bottom of the RV septum were grouped into distinct anatomic segments: superoseptum (from top to the His bundle), midseptum (from the His bundle to coronary sinus), and inferoseptum (from the coronary sinus to bottom) (Figure 1). The His bundle region was determined as the site recording the largest His bundle potential. The RV septum was further divided into three regions: a valvular region extending from the tricuspid valve to 2 cm anterior of the valve, and two equal portions between this region and the RV apex (basal and apical region), as previously reported [5], [11]. Therefore, we defined 9 distinct anatomic segments (Figure 1A). We localized the origin of PVCs/IVTs within the RV septum with fluoroscopy and by creating a electroanatomic map of the RV chamber in many patients: 1) the tip of the catheter crossed the tricuspid valve when observed in the right anterior oblique (RAO) view at the ablation site, and directed toward the RV septum when observed in the left anterior oblique (LAO) view at the ablation site; 2) When PVCs/IVTs originated from the tricuspid valvular RV septum regions, the ratio of the atrial to ventricular electrograms at the ablation site was <1, and the amplitudes of the atrial and ventricular electrograms were≧0.03 and >0.35 mV at the ablation site, respectively; 3) When PVCs/IVTs originated from the basal and apical regions of RV septum, only local ventricular electrograms and no atrial electrograms at the ablation site were recorded.

### ECG Measurements

Twelve-lead electrocardiograms recorded at a paper speed of 25 mm/s were available for all patients with PVCs/IVTs originating from the RV septum. The analysis of the ECG pattern was focusing on the following characteristics: (1) The QRS morphology of the PVCs/IVTs in all 12 leads, (2) the duration of the QRS complex, (3) the site of R-wave transition in the precordial leads (The transition zone is where the QRS complex changes from predominately negative to predominately positive and the R/S ratio becomes >1), (4) the QS pattern in the precordial leads, (5) the axis deviation. Normal QRS axis is from around −30 to +90 degrees. More negative than −30 degrees is called left axis deviation. More positive than +90 degrees is called right axis deviation.

### Pace Mapping Study

The pace mapping study was performed in the 10 control subjects. A 7-F quadripolar catheter was used. A single electrical stimulus was delivered during the end diastole in a bipolar fashion at an output just greater than the diastolic threshold from the distal electrode pair (with the distal electrode as the cathode). The catheter sites were confirmed by multi-plane fluoroscopy, and pacing was performed at these segments of the RV septum in each patient (Figure 1A). The pacing protocol was performed after a written informed consent was obtained.

### Follow-Up

After RFCA, all patients underwent a 48-hour ECG monitoring. Holter was carried out 1 week after RFCA. Patients were not given any antiarrhythmic drugs after RFCA, and underwent Doppler color echocardiography and Holter examination 3 and 6 months after RFCA. ECG, echocardiography and 24-hour ECG monitoring were performed whenever the patient had symptoms suggestive of recurrence of VAs.

### Statistical Analysis

All values were expressed as mean value ± standard deviation (SD). The continuous variables were compared with a Student’s t-test for two groups and with analysis of variance (ANOVA) for >2 groups. The categorical data were compared with a Fisher’s exact test. The sensitivity, specificity, and positive and negative predictive accuracies of the ECG characteristics to localize the site of origin of PVCs/IVTs were calculated. All tests were 2-tailed, and a value of P<0.05 was considered statistically significant.

## Results

### Study Population

Among patients with PVCs/IVTs, the incidence of VAs originating from the RV septum was 5% (total 29 patients, 18 men and 11 women; mean age 50.3±21.3 years (range 8–80 years). The clinical characteristics of the 29 patients included in the study are shown in Table 1. The past medical history was significant for hypertension in eight patients, diabetes in four patients. Symptoms consisted of palpitations (100%), presyncope (3.4%), syncope (3.4%), and chest pain (17.2%). The median duration of symptoms prior to the ablation procedure was 26.5 months (range 4–144). All patients in this study had failed prior antiarrhythmic therapy with 1.6±0.6 drugs, including amiodarone therapy in 6 patients (20.7%). No patients had a family history of sudden cardiac death, ventricular tachycardia, or cardiomyopathy. The mean left ventricular ejection fraction (LVEF) was 62.0±3.2%, and the left ventricular end-diastolic internal diameter was 45.9±2.2% mm (one patient with a markedly dilated left ventricle). No abnormalities suggestive of arrhythmogenic right ventricular cardiomyopathy were found by ECG and echocardiography and MRI and right ventricular contrast angiography in any of the patients.

**Table 1:** Baseline patient characteristics.

Patient	Age(y)	Sex	PVC count(number/24 h)	Symptoms	Symptom duration(M)	AADs used	Comorbidities	LVEF(%)	LVEDd(mm)
1	32	M	33120	palpitation	6	Beta-blocker,Propafenone	none	61	45
2	52	F	21637	palpitation	31	Beta-blocker	none	59	49
3	17	M	16283	palpitation	6	Beta-blocker	none	68	41
4	62	F	10328	palpitation	6	Beta-blocker	hypertension	60	46
5	17	M	21862	palpitation	42	Beta-blocker,Propafenone	none	62	43
6	59	F	16549	palpitation	36	Beta-blocker,Propafenone	none	59	48
7	66	F	18280	palpitation	24	Beta-blocker,Amiodarone	diabetes,hypertension	61	46
8	54	F	25390	palpitation	24	Beta-blocker	none	60	49
9	71	F	18544	palpitation	30	Beta-blocker	hypertension	62	45
10	19	M	21862	Palpitationchest pain	60	Beta-blocker,Mexiletine,Amiodarone	none	66	46
11	80	M	37618	Palpitation,presyncope	5	Beta-blocker	hypertension	57	49
12	37	F	10261	Palpitation	6	Beta-blocker	none	63	45
13	52	F	21063	Palpitation,chest pain	43	Beta-blocker,Propafenone	none	59	46
14	74	M	16802	Palpitation	6	Beta-blocker	Hypertension, diabetes	60	47
15	73	M	16538	palpitation	19	Beta-blocker,Propafenone	none	65	46
16	68	M	26982	palpitation,chest pain	10	Beta-blocker,Amiodarone	none	58	43
17	63	M	20387	palpitation	30	Beta-blocker,Propafenone	none	66	45
18	38	M	15724	palpitation	30	Beta-blocker,Mexiletine	none	67	42
19	63	M	10663	palpitation	9	Beta-blocker	none	65	44
20	19	F	32638	palpitation,chest pain	24	Beta-blocker,Propafenone	none	60	46
21	8	M	36981	palpitation, syncope	5	Beta-blocker	none	62	49
22	23	F	12220	palpitation	26	Beta-blocker,Propafenone	none	63	45
23	54	M	17063	palpitation	42	Beta-blocker,Amiodarone	none	68	45
24	70	M	19610	palpitation	36	Beta-blocker,Amiodarone	hypertension	62	46
25	32	M	11238	palpitation	18	Beta-blocker	none	67	44
26	55	F	27817	palpitationchest pain	28	Beta-blocker	diabetes	61	48
27	57	M	19627	palpitation	18	Beta-blocker,Propafenone	none	60	48
28	68	M	25317	palpitation	4	Beta-blocker	hypertension	60	45
29	76	M	11096	palpitation	144	Beta-blocker,Amiodarone	hypertension,diabetes	58	50
Mean ± SD	50.3±21.3		20466±7734		26.5±26.9			62.0±3.2	45.9±2.2

Y = years, M = Months, AADs = antiarrhythmic drugs, LVEF = left ventricular ejection fraction, LVEDd = left ventricular end-diastolic internal diameter.

### Baseline 24-Hour ECG Monitoring

The mean PVC burden during the preoperative 24 h of ambulatory Holter monitoring was 20466±7734 (range 10261–37618): 20 patients (69.1%) had single isolated PVC, 5 patients (17.2%) had ventricular couplets, 1 patient (3.4%) had non sustained monomorphic VT (defined as ≧ 3 consecutive PVCs, duration ≦ 30 s), 3 patients (10.3%) had sustained monomorphic VT (duration ≧ 30 s) (Table 1). The PVCs were monomorphic in all patients.

### Electrophysiologic Findings and Effect of RFCA

**Figure 2:**
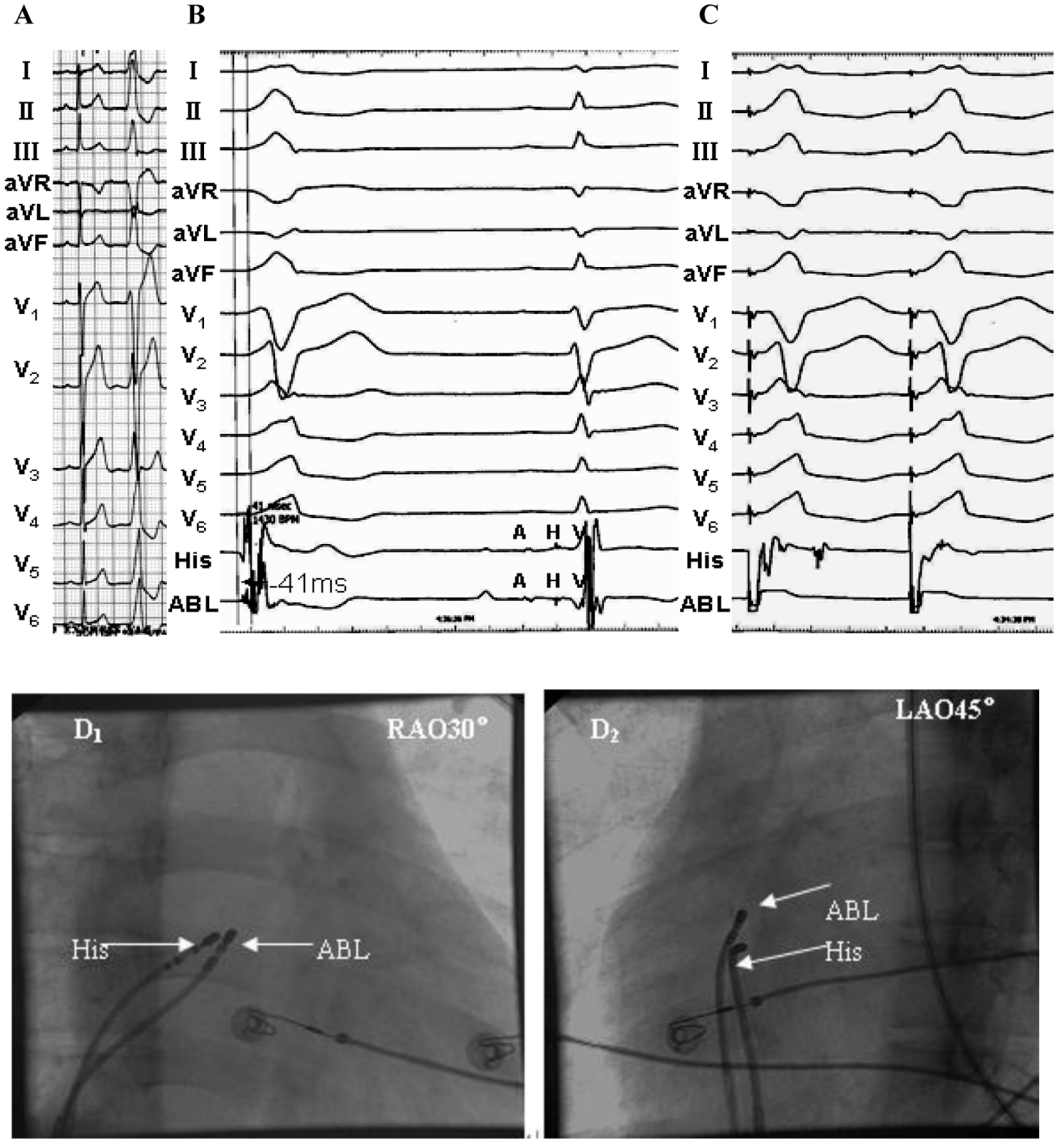
Example of a unsuccessful ablation of a premature ventricular contraction (PVC) originating from the superoseptal portion of the tricuspid valvular right ventricular region. No attempt at ablation was undertaken because the origin of the PVC was found to be near the His-bundle. (A) The surface ECG characteristic. (B) The local ventricular activation time recorded at the site that preceded the onset of the QRS complex was 41 ms. A sharp His electrogram at the site was recorded in sinus rhythm. (C) Pace map at the site. (D) The fluoroscopic position of the ablation catheter and the His-bundle catheter sites. The site of the origin of the PVC was just less than 1 cm superior to the His-bundle catheter. ABL, ablation catheter; RAO, right anterior oblique projection; LAO, left anterior oblique projection.

PVCs/IVTs arise from the RV septum in 29 patients, including 20 (69%) from the septal portion of the tricuspid valvular RV region (3 from right superoseptum, 15 from right midseptum, 2 from right inferoseptum), and 9 (31%) from the septal portion of the basal RV (1 from right superoseptum, 4 from right midseptum, 4 from right inferoseptum). No PVCs/IVTs originated from the septal portion of the apical RV. Figure 1B and Table 2 list the detailed VA origin from the RV septum. The PVCs/IVTs occurred spontaneously in 26 patients and was induced by bolus injection of isoproterenol (2 µg) in 3 patients during the electrophysiologic study. Twenty-two of 29 patients underwent the electrophysiologic study using Ensite NavX system or Carto XP system or Carto 3 system, and the remaining 7 patients using conventional mapping techniques. The local ventricular activation time recorded at successful ablation target sites that preceded the onset of the QRS complex was 30.4±5.4 ms (Table 2). Successful RFCA in 27 patients could be achieved (93.1% acute procedural success). RFCA could not be applied in the remaining 2 patients because the best pace mapping site was close to the His bundle (Figure 2). No complications occurred during the mapping or ablation procedure. An average of 3.2±1.4 (range 2–7) radiofrequency energy applications were delivered with a mean total radiofrequency energy duration of 270.9±98.4 (range 62–516)s. The mean fluoroscopy time was 8.0±2.3 (range 1.8–11.4) min. Patients have been followed-up for 11.2±8.2 (range 3–36) months. One patient had recurrent VAs after an initially effective procedure. The transthoracic echocardiography demonstrated a decreased LVEDd 6 months after effective RFCA in the patient whose LVEDd was increased prior to ablation (Patient No. 21 in Table 1). No right ventricular dilation, wall motion abnormalities, or ECG abnormalities (i.e., inverted T waves in the right precordial leads, epsilon wave) were found in any of these patients during follow-up. No patient died during follow-up.

**Table 2:** The RFCA outcome.

Patient	Origin	Mappingtechnique	EAT(ms)	Pace mapping(12 lead concordance)	Ablationoutcome	RF lesionsprior tosuccess	Proceduralcomplication	Recurrent	Follow uptime (M)
1	TV Sup	EAT+Pace	42	10	success	3	no	no	30
2	TV Sup	EAT+Pace	31	10	success	3	no	no	18
3	TV Sup	EAT+Pace	41	12	failure	–	no	–	21
4	TV Mid	EAT+Pace	28	10	success	2	no	no	4
5	TV Mid	EAT+Pace	28	12	success	3	no	no	11
6	TV Mid	EAT+Pace	24	12	success	2	no	no	11
7	TV Mid	EAT+Pace	28	12	success	2	no	no	18
8	TV Mid	EAT+Pace	30	12	success	3	no	no	12
9	TV Mid	EAT+Pace	27	12	success	5	no	no	12
10	TV Mid	EAT+Pace	32	12	success	2	no	yes	11
11	TV Mid	EAT+Pace	31	9	success	2	no	no	11
12	TV Mid	EAT+Pace	34	12	success	2	no	no	3
13	TV Mid	EAT+Pace	29	12	success	3	no	no	4
14	TV Mid	EAT+Pace	22	12	success	5	no	no	3
15	TV Mid	EAT+Pace	31	11	failure	–	no	–	6
16	TV Mid	EAT+Pace	32	11	success	4	no	no	4
17	TV Mid	EAT+Pace	32	12	success	4	no	no	6
18	TV Mid	EAT+Pace	30	11	success	7	no	no	6
19	TV Inf	EAT+Pace	30	12	success	2	no	no	24
20	TV Inf	EAT+Pace	33	12	success	3	no	no	6
21	Basal Sup	EAT+Pace	39	9	success	3	no	no	6
22	Basal Mid	EAT+Pace	20	12	success	2	no	no	12
23	Basal Mid	EAT+Pace	20	12	success	3	no	no	12
24	Basal Mid	EAT+Pace	39	12	success	3	no	no	36
25	Basal Mid	EAT+Pace	30	9	success	7	no	no	11
26	Basal Inf	EAT+Pace	30	12	success	2	no	no	12
27	Basal Inf	EAT+Pace	26	11	success	3	no	no	6
28	Basal Inf	EAT+Pace	32	12	success	4	no	no	6
29	Basal Inf	EAT+Pace	32	12	success	2	no	no	3
Total			30.4±5.4	11.3±1.04	Procedural success rate: 93.1%	3.2±1.4	Proceduralcomplication rate: 0	Recurrent rate (%): 3.7	11.2±8.2

RFCA: radiofrequency catheter ablation; EAT: earliest activation time; TV Sup: the superoseptal portion of the tricuspid valvular right ventricular region; TV Mid: the midseptal portion of the tricuspid valvular right ventricular region; TV Inf: the inferoseptal portion of the tricuspid valvular right ventricular region; Basal Sup: the superoseptal portion of the basal right ventricular region; Basal Mid: the midseptal portion of the basal right ventricular region; Basal Inf: the inferoseptal portion of the basal right ventricular region.

### ECG Characteristics

#### General characteristics

**Figure 3:**
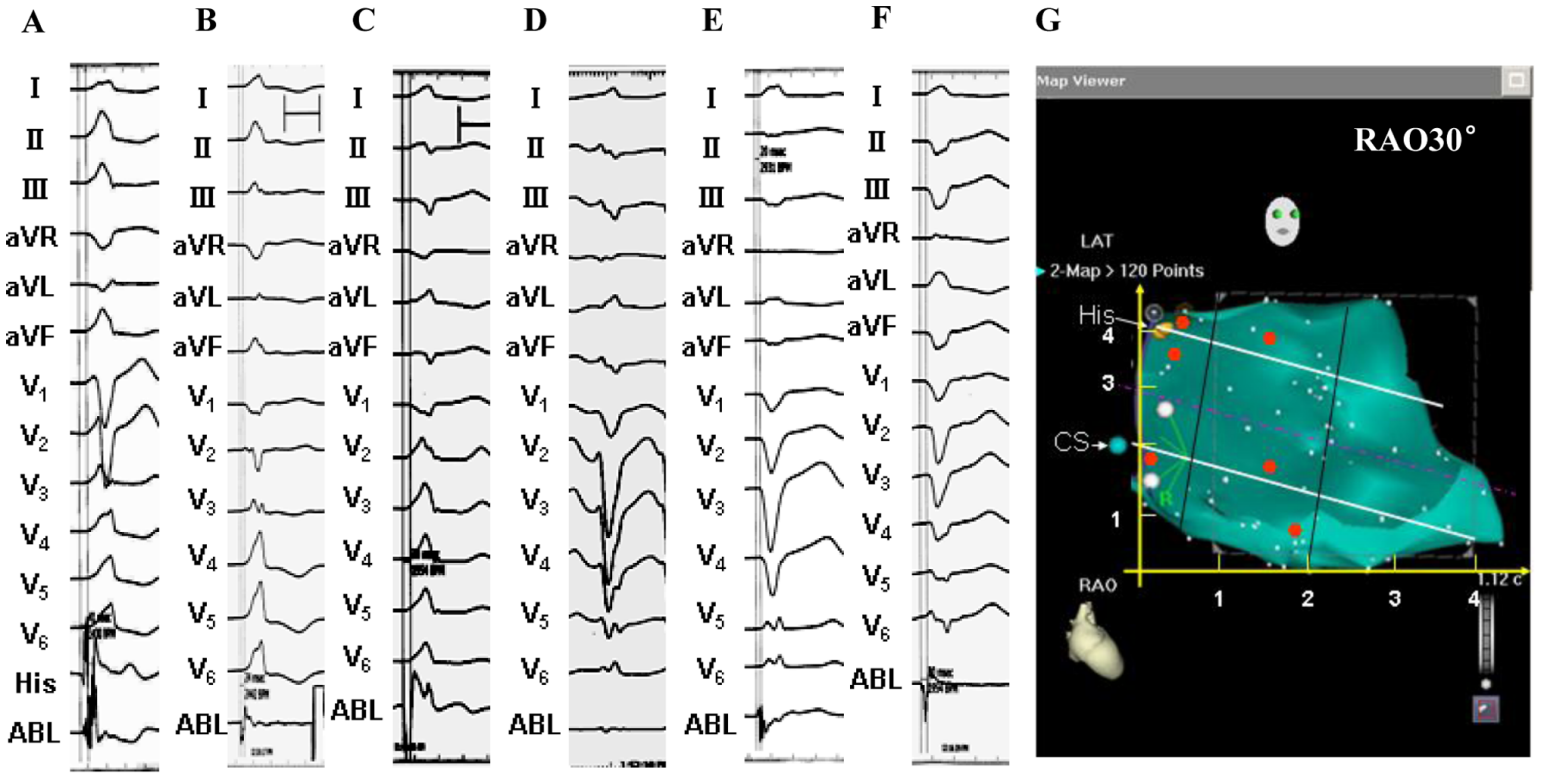
Representative 12-lead electrocardiograms of premature ventricular contractions originating from the right ventricular septum. A: the superoseptal portion of the tricuspid valvular right ventricular region; B: the midseptal portion of the tricuspid valvular right ventricular region; C: the inferoseptal portion of the tricuspid valvular right ventricular region; D: the superoseptal portion of the basal right ventricular region; E: the midseptal portion of the basal right ventricular region; F: the inferoseptal portion of the basal right ventricular region; G: Schematic right anterior oblique (RAO) view of the right ventricular septum displaying the sites of origin of the PVC/IVTs, represented by the red dots. His: largest His-bundle potential (yellow dot); CS: coronary sinus (blue dot).

**Figure 4:**
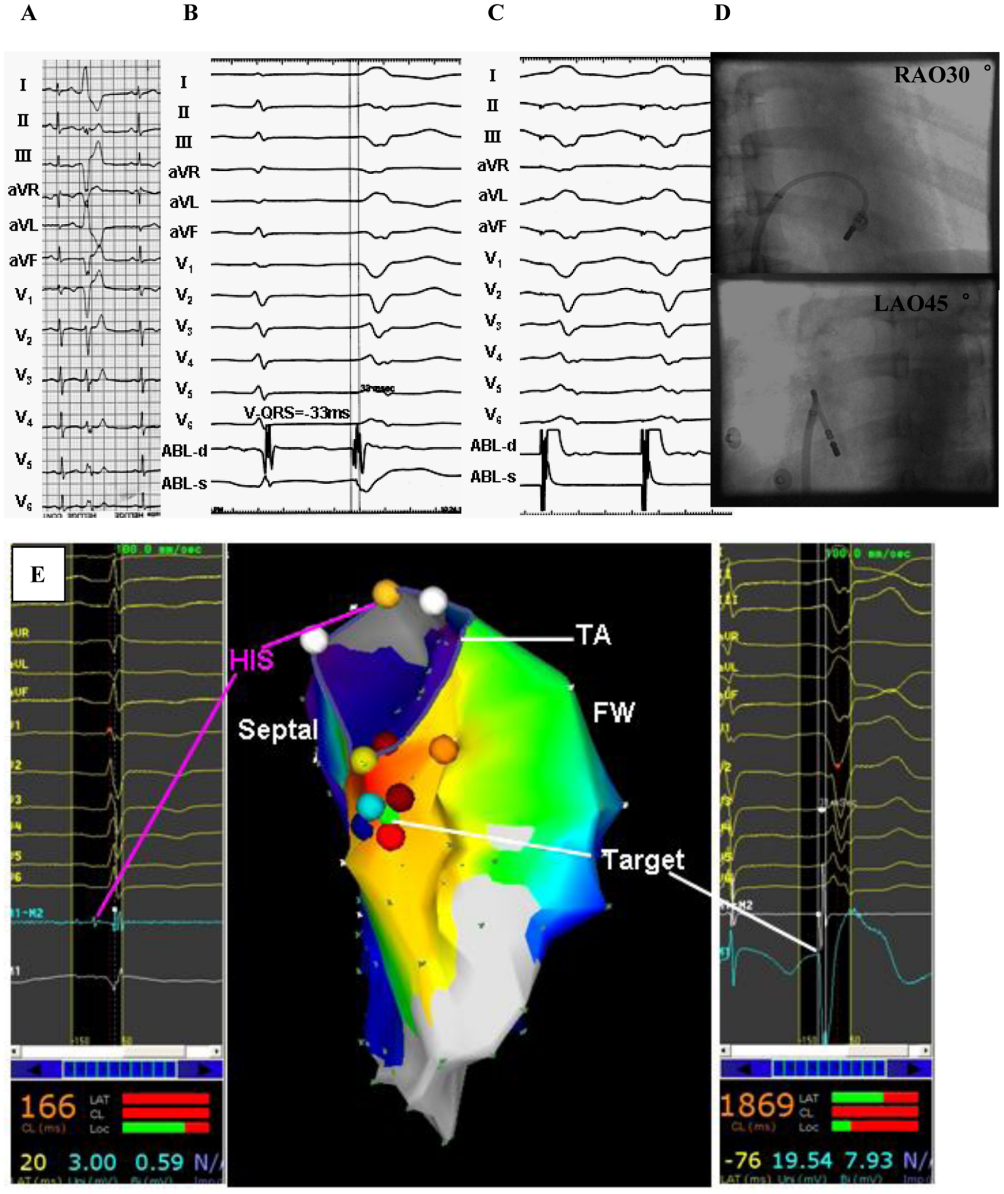
Example of a successful ablation of a premature ventricular contraction (PVC) originating from the inferoseptal portion of the tricuspid valvular right ventricular region. (A) The surface ECG characteristic. (B) The local ventricular activation time recorded at the successful ablation site that preceded the onset of the QRS complex was 33 ms. (C) Pace map at the ablation site. (D) The fluoroscopic position of the ablation catheter site. (E) Green dot indicates site of RF application under the guide of Carto 3. ABL, ablation catheter; RAO, right anterior oblique projection; LAO, left anterior oblique projection; Septal, right ventricular septum; FW, right ventricular free wall; TA, tricuspid annulus.

**Figure 5:**
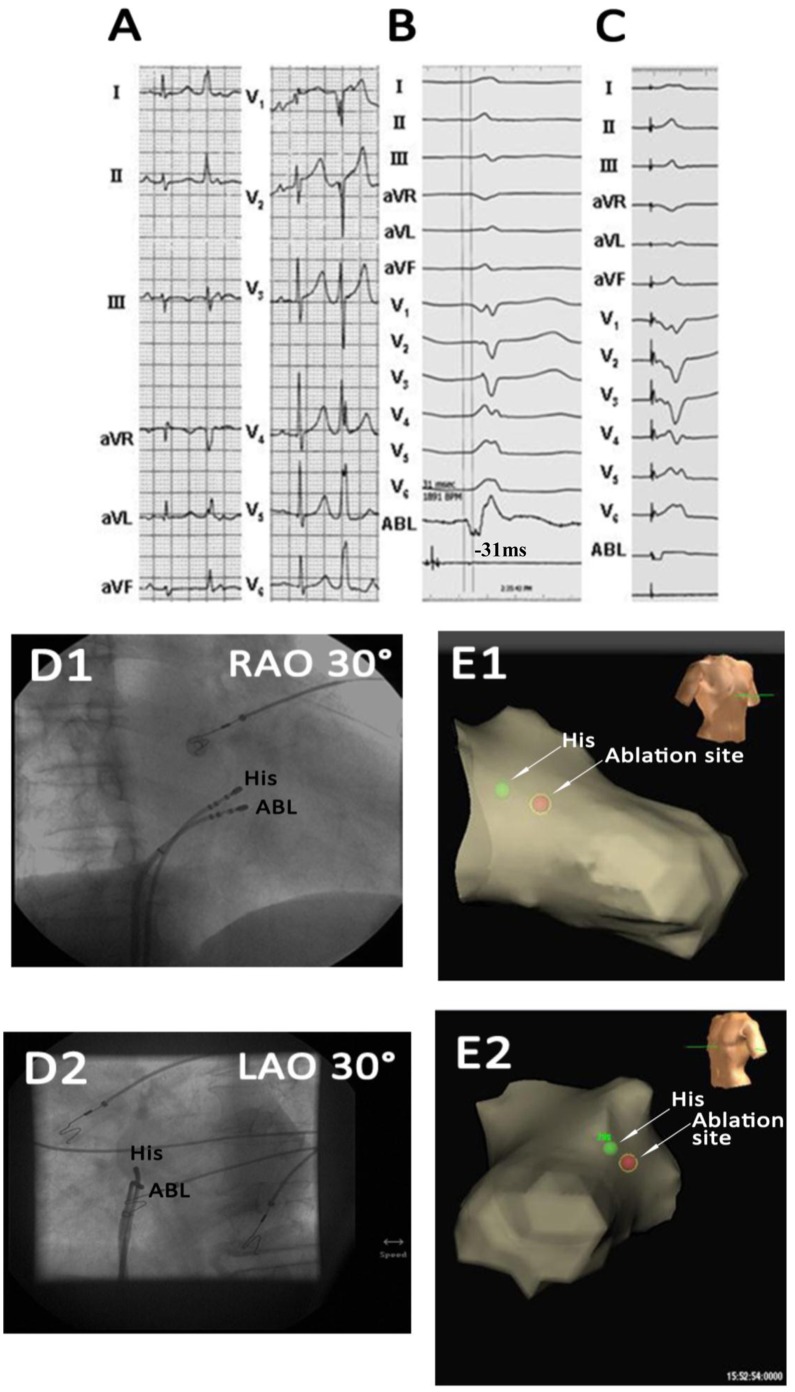
Example of a successful ablation of a premature ventricular contraction (PVC) originating from the midseptal portion of the tricuspid valvular right ventricular region. (A) The surface ECG characteristic. (B) The local ventricular activation time recorded at the successful ablation site that preceded the onset of the QRS complex was 31 ms. (C) Pace map at the ablation site. (D) The fluoroscopic position of the ablation catheter and the His-bundle catheter sites. (E) Red dot indicates site of RF application under the guide of Ensite NavX. ABL, ablation catheter; RAO, right anterior oblique projection; LAO, left anterior oblique projection.

**Figure 6:**
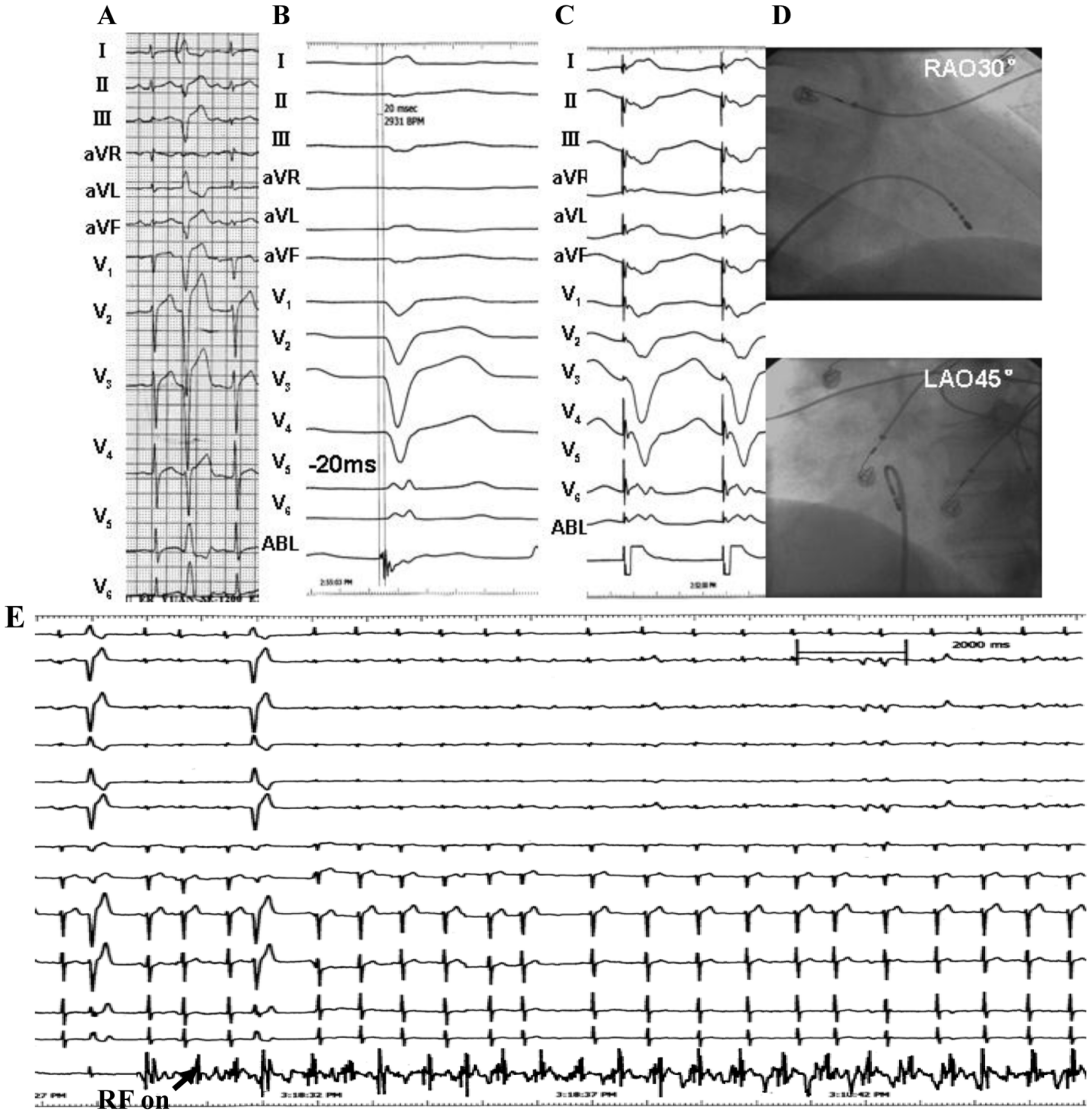
Example of a successful ablation of a premature ventricular contraction (PVC) originating from the midseptal portion of the basal right ventricular region. (A) The surface ECG characteristic. (B) The local ventricular activation time recorded at the successful ablation site that preceded the onset of the QRS complex was 20 ms. (C) Pace map at the ablation site. (D) The fluoroscopic position of the ablation catheter site. (E) Termination of PVCs within 3 seconds during RF application at the site. ABL, ablation catheter; RAO, right anterior oblique projection; LAO, left anterior oblique projection.

**Figure 7:**
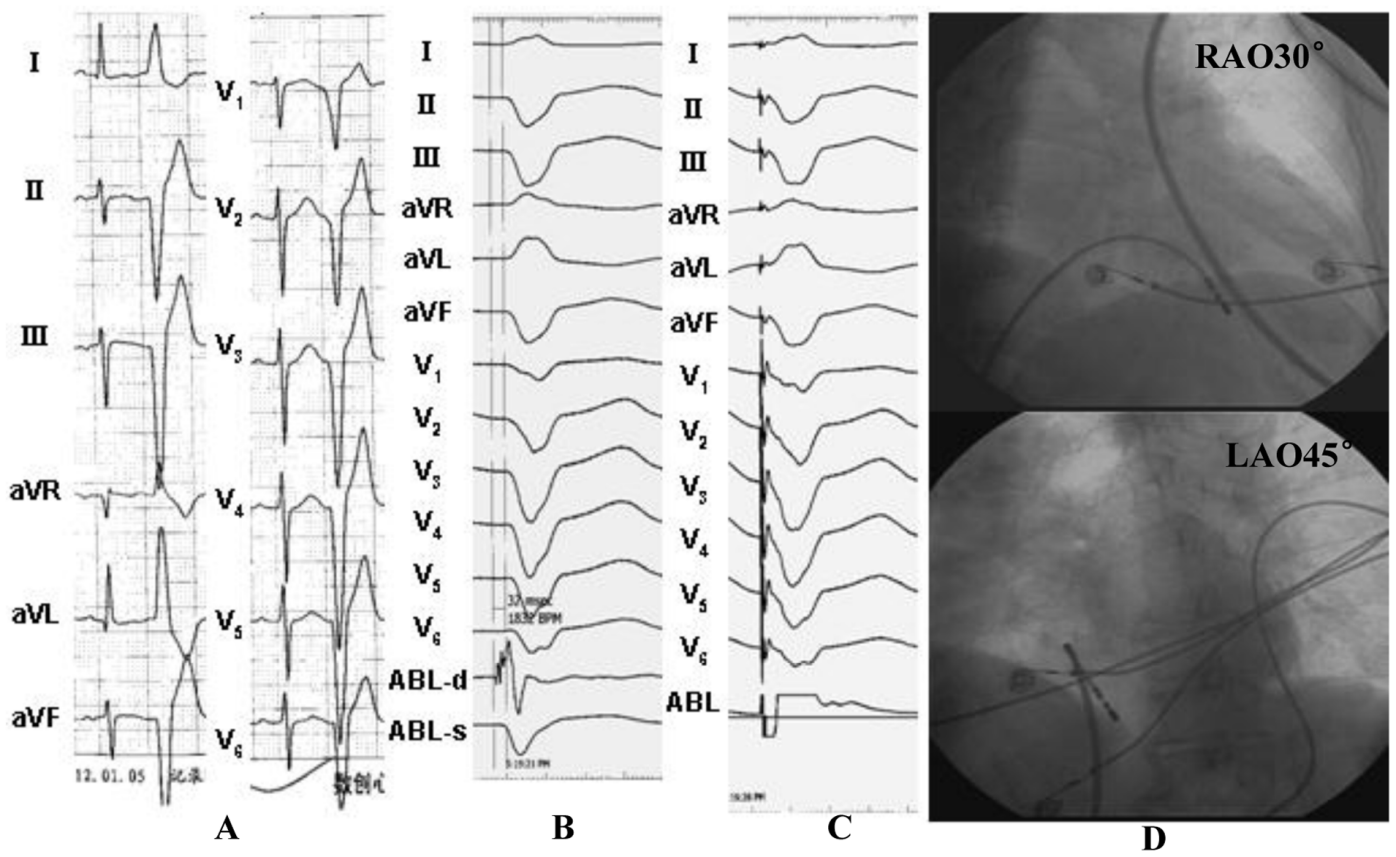
Example of a successful ablation of a premature ventricular contraction (PVC) originating from the inferoseptal portion of the basal right ventricular region. (A) The surface ECG characteristic. (B) The local ventricular activation time recorded at the successful ablation site that preceded the onset of the QRS complex was 32 ms. (C) Pace map at the ablation site. (D) The fluoroscopic position of the ablation catheter site. ABL, ablation catheter; RAO, right anterior oblique projection; LAO, left anterior oblique projection.

All PVCs/IVTs arising from the RV septum had an rS or QS pattern in lead V1 and displayed a left bundle branch block morphology (Figures 2–7 and Table 3). All PVCs/IVTs showed a monophasic R pattern in lead I, 86.2% (25/29) PVCs/IVTs had a QS or qs pattern in lead aVR, and 79.3% (23/29) PVCs/IVTs had a monophasic R pattern in lead aVL. The duration of the QRS complex of the PVCs/IVTs was132±10 (range 110–150) ms. The precordial R-wave transition occurred in or earlier than lead V3 in 14 patients, and later than lead V3 in 15 patients (earlier than lead V2 in 2 patients, between lead V2 and V3 in 6 patients, in lead V3 in 6 patients, between lead V3 and V4 in 6 patients, between lead V4 and V5 in 4 patients, between lead V5 and V6 in 2 patients, in lead V6 in 1 patient, later than lead V6 in 2 patients).

**Table 3:** 12-lead ECG characteristics of PVCs/IVTs originating from the right ventricular septum.

Pt	origin	QRS axis	QRS complex morphology												TransitionZone	QRS duration(ms)
			V1	V2	V3	V4	V5	V6	I	aVR	aVL	II	III	aVF		
1	TV Sup	left	rS	rS	Rs	R	R	R	R	QS	R	R	Rs	R	V2∼V3	130
2	TV Sup	normal	rS	rS	RS	R	R	R	R	QS	qr	R	Rs	R	V3	130
3	TV Sup	normal	rS	RS	Rs	R	R	R	R	QS	m	R	Rs	R	V2∼V3	150
4	TV Mid	left	QS	QS	rS	R	R	R	R	QS	qR	Rs	rS	rS	V3∼V4	130
5	TV Mid	left	QS	Rs	Rs	R	R	R	R	QS	R	RS	RS	RS	V2∼V3	120
6	TV Mid	normal	QS	rS	RS	R	R	R	R	QS	R	R	rs r’	R	V2∼V3	140
7	TV Mid	left	QS	RS	Rs	R	R	R	R	QS	r	R	rs	Rs	V2∼V3	140
8	TV Mid	left	QS	QS	Rs	R	R	R	R	QS	qr	R	Rs	Rs	V3∼V4	130
9	TV Mid	left	QS	rS	rS	Rs	R	R	R	QS	R	R	qs	rs	V3∼V4	130
10	TV Mid	left	QS	QS	RS	R	R	R	R	QS	R	R	qs	rs	V3∼V4	130
11	TV Mid	left	QS	QS	RS	R	R	R	R	QS	R	R	qRs	qRs	V3	110
12	TV Mid	left	QS	rS	rS	R	R	R	R	QS	R	R	rS	rs	V3∼V4	150
13	TV Mid	left	QS	rS	Rs	R	R	R	R	QS	R	R	QS	rs	V2∼V3	120
14	TV Mid	left	QS	rS	RS	R	R	R	R	QS	R	Rs	rS	rS	V3	140
15	TV Mid	left	QS	rS	RS	R	R	R	R	QS	R	Rs	rS	rS	V3	130
16	TV Mid	normal	QS	QS	rS	R	R	R	R	QS	qR	R	Rs	Rs	V3∼V4	120
17	TV Mid	left	QS	rS	RS	R	R	R	R	QS	R	R	rs	Rs	V3	140
18	TV Mid	left	QS	Rs	R	R	R	R	R	QR	R	Rs	rS	rS	V1∼V2	140
19	TV Inf	normal	QS	R	R	R	R	R	R	QS	R	rs	QS	rs	V1∼V2	150
20	TV Inf	left	QS	rS	rs	rs	rs	rs	R	QS	R	rs	rS	rS	V3	120
21	Basal Sup	left	rS	rS	rS	rS	rS	R	R	QS	R	rs	rS	rS	V5∼V6	130
22	Basal Mid	normal	QS	QS	QS	rS	R	R	R	QS	qs	R	R	R	V4∼V5	120
23	Basal Mid	left	QS	QS	QS	rS	R	R	R	qs	R	rS	QS	rS	V4∼V5	120
24	Basal Mid	left	QS	QS	rS	rS	R	R	R	qs	R	rS	rS	rS	V4∼V5	120
25	Basal Mid	left	QS	QS	QS	QS	rs	rs	R	QS	R	rS	rS	rS	V5∼V6	140
26	Basal Inf	left	QS	QS	QS	rS	rS	rS	R	QS	R	rS	QS	QS	> V6	130
27	Basal Inf	left	rS	rS	rS	rS	rS	rs	R	qr	R	rS	rS	rS	V6	140
28	Basal Inf	left	QS	QS	QS	QS	QS	QS	R	R	R	QS	QS	QS	> V6	140
29	Basal Inf	left	QS	rS	rS	rS	R	R	R	qr	R	rS	rS	rS	V4∼V5	140

Capital letters (Q,R,S) refer to relatively high-amplitude waves (>5 mm). Conversely, lowercase letters (q,r,s) refer to relatively low-amplitude waves (<5 mm).

#### Comparison between PVCs/IVTs arising from the tricuspid valvular and basal RV septum

**Figure 8:**
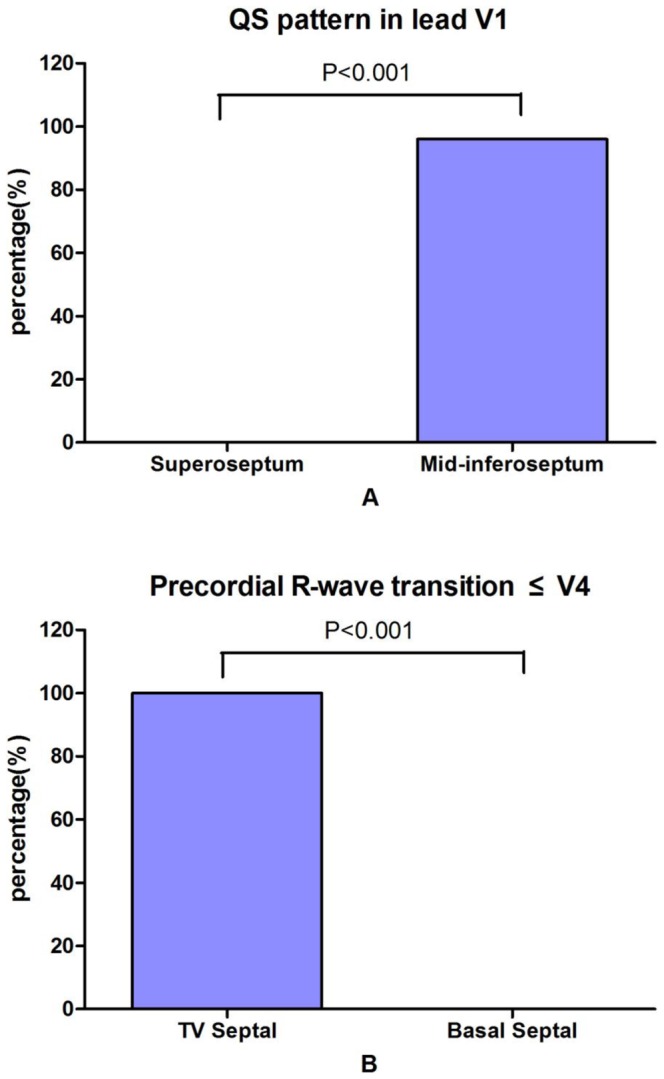
Differences in (A) QS pattern in lead V1 between PVCs/IVTs arising from the RV superoseptum and mid-inferoseptum and (B) precordial R-wave transition occurring by lead V4 between PVCs/VTs arising from the tricuspid valvular and basal right ventricular septum.

There was no significant difference in QRS duration between the PVCs/IVTs arising from the tricuspid valvular and basal RV septum. The precordial R-wave transition occurred in lead V4 or earlier (≦V4) in all of the PVCs/IVTs arising from the tricuspid valvular RV septum, and all patients with the PVCs/IVTs arising from the basal RV septum had a later precordial R-wave transition (>V4) (Figure 8 and Table 4). The earlier precordial R-wave transition (≦V4) has a sensitivity of 100%, a specificity of 100%, a negative predictive value of 100%, and a positive predictive value 100% to predict the tricuspid valvular RV septal origin of PVCs/IVTs (Table 5).

**Table 4:** Comparison of ECG characteristics between PVCs/VTs arising from the tricuspid valvular and basal right ventricular septum, between PVCs/IVTs arising from the RV superoseptum and mid-inferoseptum.

Group	n	QS pattern in precordial leads				Precordial R-wave transition			
		V1	V2	V3	V4	≤V4	V4–V5	V5–V6	>V6
TV Septal	20	17	5	0	0	20**	0	0	0
Basal Septal	9	7	7	5	2	0	4	2	3
Superoseptum	4	0	0	0	0	3	0	1	0
Mid-inferoseptum	25	24*	12	5	2	17	4	1	3

TV Septal: the tricuspid valvular right ventricular septum; Basal Septal: the basal right ventricular septum;

* p<0.001 Superoseptum versus Mid-inferoseptum,

** p<0.005 TV Septal versus Basal Septal.

**Table 5:** The sensitivity, specificity, negative predictive value (NPV) and positive predictive value (PPV) to identify the precise origin of PVCs/IVTs from the right ventricular (RV) septum.

ECG variables	Sensitivity (%)	Specificity (%)	NPV (%)	PPV (%)
Precordial R-wave transition ≤V4 in patients with PVCs/IVTs arisingfrom the tricuspid valvular RV septum	100	100	100	100
QS pattern in lead V1 in patients with PVCs/IVTs arising from theRV mid-inferoseptum	96.00	100	80.00	100

#### Comparison between PVCs/IVTs arising from the RV superoseptum and mid-inferoseptum

PVCs/IVTs arise from the RV septum in 29 patients, including 4 (13.8%) from the RV superoseptum and 25 (86.2%) from the RV mid-inferoseptum. There was no significant difference in QRS complex duration between the PVCs/IVTs arising from the RV superoseptum and mid-inferoseptum. A QS pattern in lead V1 was observed in 96% (24/25) of the PVCs/IVTs arising from the RV mid-inferoseptum, and an rS pattern in lead V1 was observed in all of PVCs/IVTs arising from the RV superoseptum (Figure 8 and Table 4). The QS pattern in lead V1 has a sensitivity of 96%, a specificity of 100%, a positive predictive value 100%, and a negative predictive value of 80% to predict the RV mid-inferoseptal origin of PVCs/IVTs (Table 5). When the origin of the PVCs/IVTs shifted from inferoseptum to midseptum to superoseptum of the RV (Figure 3): R wave amplitude increased and S wave amplitude decreased in leads II, III, aVF and V2–V6; R wave amplitude decreased in lead aVL; QS amplitude increased in lead aVR.

#### Comparison among PVCs/IVTs arising from the RV septum, RV free wall and RV outflow tract (RVOT)

**Figure 9:**
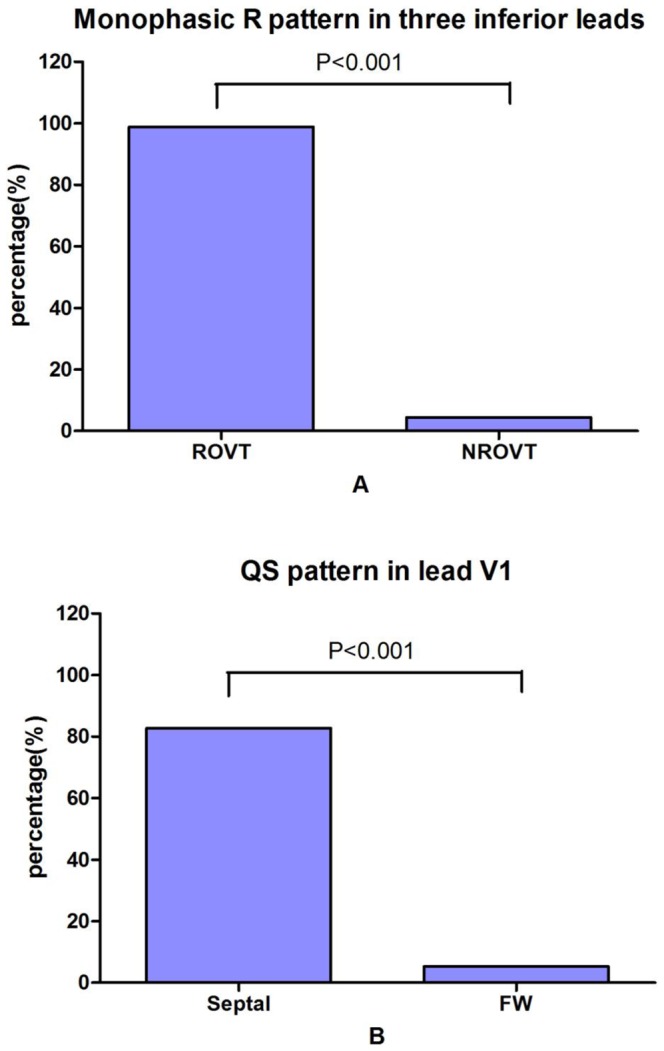
Differences in (A) Monophasic R pattern in three inferior leads between PVCs/IVTs arising from the right ventricular outflow tract (RVOT) and not arising from the right ventricular outflow tract (NRVOT) and (B) QS pattern in lead V1 between PVCs/VTs arising from the RV septum (Septal) and free wall (FW).

**Figure 10:**
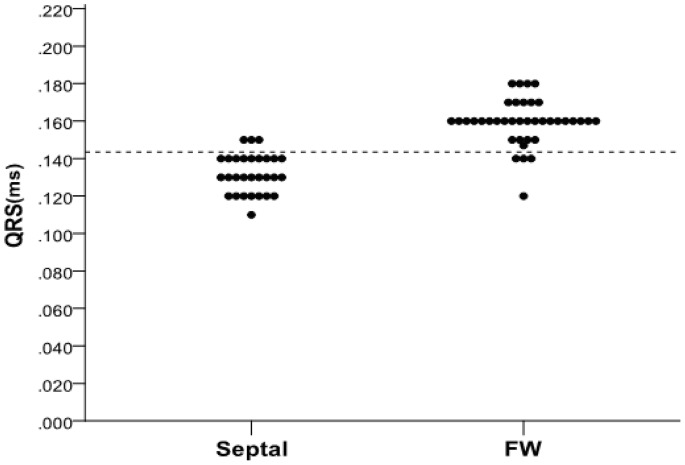
Distribution of QRS duration in patients with idiopathic PVC/VTs arising from the right ventricular septum (Septal) and free wall (FW). The QRS duration of ≦143.5 suggested the right ventricular septum origin with high sensitivity and specificity.

The QRS duration in PVCs/IVTs arising from the RV septum were significantly shorter compared with the arrhythmias arising from the RV free wall (132±10 ms VS 159±12 ms, P<0.001 ) and RVOT (132±10 ms VS 142±14 ms, P<0.001) (Table 6). Right axis deviation was noted in 87.4% (76/87) PVCs/IVTs in the RVOT group, and left axis deviation was noted in 79.3% (23/29) PVCs/IVTs in the RV septum group and in 71.1% (27/38) PVCs/IVTs in the RV free wall group. QS pattern and rS pattern in lead V1 were observed in 82.8% (24/29) and 17.2% (5/29) of the PVCs/IVTs arising from the RV septum, versus 2.3% (2/87) and 97.7% (85/87) of the PVCs/IVTs arising from the RVOT (P<0.001 for both) and 5.3% (2/38) and 94.7% (36/38) of PVCs/IVTs arising from the RV free wall (P<0.001 for both) (Figure 9 and Table 6). In the RVOT group, 86/87 (98.9%) had a monophasic R pattern in all three inferior leads (II, III, aVF) (Figure 9 and Table 6). Only 1/29 (3.5%) in the RV septum group and 2/38 (5.3%) in the RV free wall group had a monophasic R pattern in all inferior leads (98.9% VS 3.5%, 98.9% VS 5.3%; both P<0.001) (Figure 9 and Table 6). The monophasic R pattern in all inferior leads has a sensitivity of 98.9%, a specificity of 95.5%, a positive predictive value 96.6%, and a negative predictive value of 100.00% to predict the RVOT origin of PVCs/IVTs (Table 7). The QS pattern in lead V1 has a sensitivity of 82.8%, a specificity of 94.7%, a positive predictive value 92.3%, and a negative predictive value of 87.8%, and the QRS duration of ≦143.5 ms has a sensitivity of 89.7%, a specificity of 89.5%, a positive predictive value 86.7%, a negative predictive value of 91.9%and to predict the RV septum origin of PVCs/IVTs (Figure 10 and Table 7).

**Table 6:** Comparison of ECG characteristics among PVCs/VTs arising from the right ventricular (RV) septum, RV free wall and RV outflow tract (RVOT).

Origin	n	QRS duration (ms)	QRS axis			Monophasic R pattern in three inferior leads	QRS pattern in lead V1		Precordial R-wave transition		
			Left	Right	Normal		rS	QS	<V3	V3	>V3
Septum	29	132.1±10.5	23	0	6	1	5	24	8	6	15
Free wall	38	159.4±12.1^a^	27	1	10	2	36 a	2 a	0	6	31
RVOT	87	142.4±14.4a b	0	76 a b	11	86 a b	85 a	2 a	10	35	37

a p<0.001 versus RV Septum,

b p<0.001 versus RV Free wall.

**Table 7:** The sensitivity, specificity, negative predictive value (NPV) and positive predictive value (PPV) to identify the precise origin of PVCs/IVTs arising from the right ventricular outflow tract (RVOT) and not arising from the RVOT (NRVOT) and PVCs/VTs arising from the RV septum and free wall.

ECG variables	Sensitivity(%)	Specificity(%)	NPV(%)	PPV(%)
Monophasic R pattern in all three inferior leads in patients with PVCs/IVTs arising from the RVOT	98.9(86/87)	95.5(64/67)	100.00(64/65)	96.6(86/89)
QS pattern in lead V1 in patients with PVCs/IVTs arising from the RV septum	82.8(24/29)	94.7(36/38)	87.8(36/41)	92.3(24/26)
QRS duration of ≦143.5 ms in patients with PVCs/IVTs arising from the RV septum	89.7(26/29)	89.5(34/38)	91.9(34/37)	86.7(26/30)

### Pace Mapping Study

**Figure 11:**
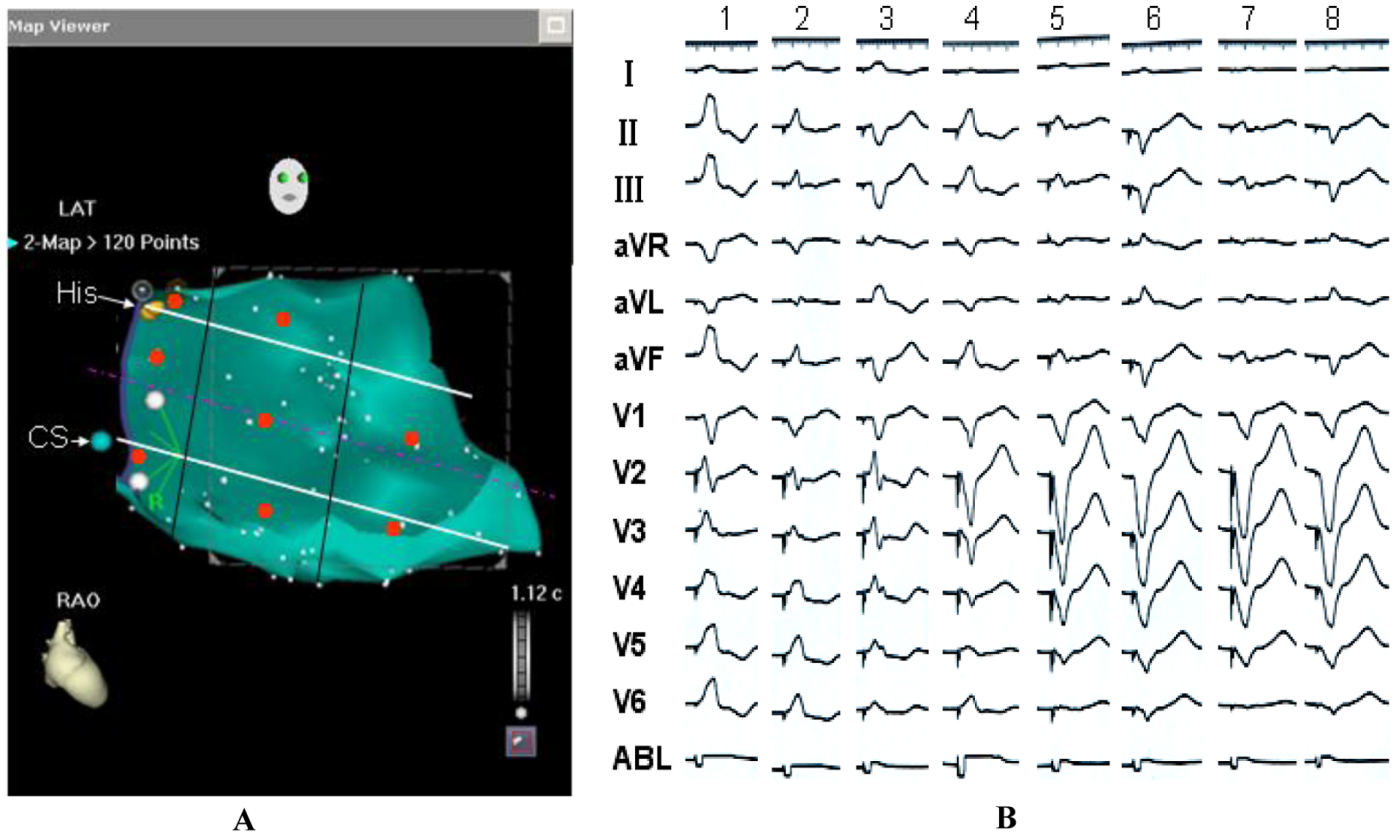
Pace mapping from the different regions of the right ventricular septum. A: Schematic right anterior oblique (RAO) view of the right ventricular septum displaying the different pacing sites represented by the red dots; B: The characteristics of the QRS morphology during pacing at the different regions of the right ventricular septum. 1, the superoseptal portion of the tricuspid valvular right ventricular region; 2, the midseptal portion of the tricuspid valvular right ventricular region; 3, the inferoseptal portion of the tricuspid valvular right ventricular region; 4: the superoseptal portion of the basal right ventricular region; 5: the midseptal portion of the basal right ventricular region; 6: the inferoseptal portion of the basal right ventricular region; 7, the midseptal portion of the apical right ventricular region; 8, the inferoseptal portion of the apical right ventricular region.

Because it was difficult to position the catheter in the superoseptal portion of the apical RV region, pace mapping at the region was not performed. The pace mapping was performed at the other 8 segments of the RV septum. The characteristics of the QRS morphology during pacing from the RV septum in 10 subjects were almost identical with those of the PVCs/IVTs arising from the RV septum (Figure 11). There existed some distinctive ECG characteristics during pacing in 10 subjects (Figure 11): 1) During pacing from superoseptum to inferoseptum of RV and from tricuspid valvular RV septum to apical RV septum, R wave amplitude was more lessening and S wave amplitude was more increasing in leads II, III, aVF and V4–V6. 2) During pacing from superoseptum to inferoseptum of RV, R wave amplitude was more increasing and Q wave amplitude was more lessening in lead aVL. 3) A rS pattern in lead V1 was observed only when pacing at the superoseptal portion of the tricuspid valvular RV region, and a QS pattern in lead V1 was always recorded when pacing at the other sites of the RV septum. During pacing from tricuspid valvular RV septum to apical RV septum, the number of precordial leads displaying the QS pattern was more increasing, and the number of precordial leads displaying the R or RS pattern was more decreasing (Figure 11).

## Discussion

### Main Findings

The study demonstrated for the first time that 5% of PVCs/IVTs had an origin at the RV septum, and that the septal portion of the tricuspid valvular RV region was the preferential site of origin, but the site of origin also could be located at the septal portion of the basal RV region. We note different ECG characteristics of PVCs/IVTs originating from the different portions of the RV septum which were confirmed by the pace mapping study and demonstrate that RFCA can be performed safely with good long-term success in preventing symptomatic VAs.

### Site-specific ECG Characteristics and their Proposed Mechanisms

Prior studies described VAs originating from the tricuspid annulus and RV septum near the His-bundle in patients without structural heart disease [7]–[10]. We and other investigators have recently demonstrated that QRS duration, R-wave transition in the precordial leads, QS pattern in lead V1, and polarity of the QRS complex in the inferior leads were useful to distinguish between PVCs/IVTs originating from the septal portion and free wall of tricuspid annulus [8], [9]. A monophasic tall R pattern in lead I, low R wave in leads II and aVF, particularly lower R wave in lead III than in lead II, R wave in lead aVL, relatively narrow QRS duration, QS pattern in lead V1, and tall R wave in V5 and V6 suggest the PVCs/IVTs origin from the RV septum near the His-bundle [10]. Although several studies have reported the PVCs/IVTs arising from the tricuspid annulus and RV septum near the His-bundle, none have systemically determined the prevalence and ECG characteristics of PVCs/IVTs arising from the different sites of the RV septum, based on as many patients as in this study. In the present study, the relatively early precordial R wave transition by lead V4 help us to differentiate the tricuspid valvular septum from the basal RV septal site of origin. Because the origin of VAs in the tricuspid valvular septum was located in the more posterior portion of the RV than that in the basal septum of the RV, more distant from the precordial electrodes, the myocardium near the focus was depolarized in a direction more toward these electrodes. This could account for the earlier precordial R-wave transition (≦V4) in the PVCs/IVTs arising from the tricuspid valvular septum than the basal RV septum. In the present study, another interesting finding was that when the origin of the PVCs/IVTs shifted from inferoseptum to midseptum to superoseptum of the RV, R wave amplitude increased and S wave amplitude decreased in leads II, III, aVF and V2–V6, R wave amplitude decreased in lead aVL, and QS amplitude increased in lead aVR. Because the origin of the PVCs/IVTs arising from the RV inferoseptum was located on the right inferior side of the heart, the myocardium would be depolarized in a direction toward the anode of leads aVL, away from the inferior leads, which might account for the findings: lower R wave and greater S wave amplitude in the inferior leads, and greater R wave amplitude in the lead aVL; the origin of the PVCs/IVTs arising from the RV superoseptum was located on the right superior side of the heart, the myocardium would be depolarized in a direction toward the inferior leads, away from the lead aVR and aVL, which might account for the findings: greater R wave and smaller S wave amplitude in the inferior leads, greater QS amplitude in lead aVR, smaller R amplitude in lead aVL. In the present study, the QS pattern in lead V1 and QRS duration were found to be useful to distinguish PVCs/IVTs origin in the RV septum or the RV free wall. Because the magnitude of the initial ventricular force is expected to be much greater in the left ventricle than in the RV, the mean initial QRS vector would be directed to the left posterior and away from the electrode of lead V1 on the horizontal plane, which might predominantly result in a QS pattern in PVCs/IVTs arising from the RV septum. When the VAs focus is located at the RV septum, both ventricles would be activated almost simultaneously, which might result in a shorter QRS duration. In the present study, s or S wave in the inferior leads, particularly in lead III was observed more often PVCs/IVTs arising from the RV septum than that arising from the RVOT. In contrast, the monophasic R pattern in all of the inferior leads was observed more often PVCs/IVTs arising from the RVOT than that arising from the RV septum. Because the RV septum is positioned to the right and inferior to the RVOT. Therefore, in PVCs/IVTs arising from the RV septum, it would be directed more to the left and superiorly than in PVCs/IVTs arising from the RVOT, which may account for the rarities of a monophasic R pattern in all of the inferior leads in PVCs/IVTs arising from the RV septum.

### Radiofrequency Ablation

Previous studies have shown that PVCs/IVTs originating from the septal portion of tricuspid annulus and RV septum near the His-bundle can be successfully ablated [7]–[10]. In the present study, we demonstrated that RFCA was effective for eliminating PVCs/IVTs arising from the RV septum. Twenty-seven of 29 patients with PVCs/IVTs arising from the RV septum were successfully ablated (93.1% acute success). In the remaining 2 patients no attempt at ablation was undertaken because the origin of the PVCs/IVTs was found to be parahisian. One patient had recurrent ventricular arrhythmia after successful RFCA during a mean follow-up period of 11.2 months. No significant complications were observed in our patient group confirming the safety of the procedure. Although clinical application of RF energy in the RV septum has proven relatively safe, much care must be taken to avoid the complications, such as atrioventricular node conduction block.

### Study Limitations

No abnormalities suggestive of arrhythmogenic right ventricular cardiomyopathy were found by ECG and echocardiography and MRI and right ventricular contrast angiography in any of the patients with the RV septal VAs in the present study. No ECG and echocardiography changes suggestive of arrhythmogenic right ventricular cardiomyopathy were seen during follow up. However, a signal-averaged ECG or endomyocardial biopsy was no performed. Therefore, we could not have completely excluded the possibility of a concealed form of arrhythmogenic right ventricular cardiomyopathy. However, we believe the data presented strongly suggests that the arrhythmias described were truly idiopathic. To increase the accuracy of our study, our results need to be confirmed in additional long-term follow-up.

### Conclusions

We identified a group of patients with PVCs/IVTs arising from the RV septum. ECG characteristics of PVCs/VTs originating from the different portions of the RV septum are different, and can help regionalize the origin of these arrhythmias. RFCA at the RV septum was effective and safe for the PVCs/IVTs.
